# Photometric and Colorimetric Assessment of LED Chip Scale Packages by Using a Step-Stress Accelerated Degradation Test (SSADT) Method

**DOI:** 10.3390/ma10101181

**Published:** 2017-10-16

**Authors:** Cheng Qian, Jiajie Fan, Jiayi Fang, Chaohua Yu, Yi Ren, Xuejun Fan, Guoqi Zhang

**Affiliations:** 1 School of Reliability and Systems Engineering, Beihang University, Beijing 100191, China; cqian@sklssl.org; 2 College of Mechanical and Electrical Engineering, Hohai University, Changzhou 213022, China; yuchaohua123@hhu.edu.cn (C.Y.); jay.fan@connect.polyu.hk (J.F.);; 3 Changzhou Institute of Technology Research for Solid State Lighting, Changzhou 213161, China; 4 College of Mathematics and Physics, Changzhou University, Changzhou 213164, China; 5 Department of Mechanical Engineering, Lamar University, Beaumont, TX 77710, USA; xfan@lamar.edu; 6 EEMCS Faculty, Delft University of Technology, Delft 2628, The Netherlands; g.q.zhang@tudelft.nl

**Keywords:** light-emitting diode, chip scale package, accelerated aging, step stress test, reliability qualification

## Abstract

By solving the problem of very long test time on reliability qualification for Light-emitting Diode (LED) products, the accelerated degradation test with a thermal overstress at a proper range is regarded as a promising and effective approach. For a comprehensive survey of the application of step-stress accelerated degradation test (SSADT) in LEDs, the thermal, photometric, and colorimetric properties of two types of LED chip scale packages (CSPs), i.e., 4000 °K and 5000 °K samples each of which was driven by two different levels of currents (i.e., 120 mA and 350 mA, respectively), were investigated under an increasing temperature from 55 °C to 150 °C and a systemic study of driving current effect on the SSADT results were also reported in this paper. During SSADT, junction temperatures of the test samples have a positive relationship with their driving currents. However, the temperature-voltage curve, which represents the thermal resistance property of the test samples, does not show significant variance as long as the driving current is no more than the sample’s rated current. But when the test sample is tested under an overdrive current, its temperature-voltage curve is observed as obviously shifted to the left when compared to that before SSADT. Similar overdrive current affected the degradation scenario is also found in the attenuation of Spectral Power Distributions (SPDs) of the test samples. As used in the reliability qualification, SSADT provides explicit scenes on color shift and correlated color temperature (CCT) depreciation of the test samples, but not on lumen maintenance depreciation. It is also proved that the varying rates of the color shift and CCT depreciation failures can be effectively accelerated with an increase of the driving current, for instance, from 120 mA to 350 mA. For these reasons, SSADT is considered as a suitable accelerated test method for qualifying these two failure modes of LED CSPs.

## 1. Introduction

With a wide range of applications of LED products in the lighting market, the reliability of the phosphor converted white LED (pc-white LED) has become a global spread hot research [1,2,3]. A white LED with high reliability is expected to have a lower luminous flux degradation and color shift under a long-term operation time. Although some fundamental and advanced algorithms were developed to assist people to understand more about the essence of reliability of pc-white LEDs, unfortunately, the traditional reliability evaluation test methods still require a test time of at least 6000 h, which is definitely a too long time for LED manufacturers [4,5,6,7,8,9,10,11,12,13]. Therefore, fast and effective test methods are urgently needed with time and cost considerations [14,15]. 

In order to shorten the test duration, the constant stress accelerated degradation tests (CSADTs) under manifold uni/multi-environmental overstress conditions are developed for pc-white LEDs and the relevant products [2,16]. Through the deep investigation on the degradation characteristics of LEDs, the accelerated loadings could be anyone among high temperature [17], high moisture [18], and a hybrid combination of both [19,20,21,22]. However, the selection of practical accelerated loadings always meets a dilemma between accuracy and universality. Due to the diversity of the failures existing in LED products, a complex coupled stress might be a good accelerated test solution for some LED products but totally invalid for many others. Improvement of the universality is often achieved by reducing the complexity of the accelerated loadings. For a general application, C. Qian et al. developed a CSADT method to reduce the reliability test period from 6000 h to 2000 h for LED luminaires and lamps [17]. Via a boundary curve theory, they proved that the qualification results obtained from the 6000 h test data under 25 °C and 1500 h test data under 55 °C are comparable to each other for a majority of LED lighting products.

Step stress accelerated degradation test (SSADT) is another commonly utilized test plan for lifetime evaluation of high reliability products [23]. When combined with stochastic degradation models such as Gamma process and Wiener process, and statistical luminous flux distributions such as Gaussian distribution, Weibull distribution and Lognormal distribution, SSADT is capable to provide more efficient lifetime evaluation test plan with a smaller sample size and less test time for LED products, as compared to CSADT [24,25,26]. This makes the optimization of the SSADT test strategy become a very hot research topic. Han proposed three different kinds of design criteria (i.e., C/D/A-optimal design criteria) for optimizing the increment step and test duration of each stress level in the SSADT with a constraint limit on the test cost [25]. Among these three design plans, the C-optimal design was applied to minimize the lifetime variability, D-optimal design was applied maximize the joint precision of estimators of the lifetime model parameters, whereas A-optimal design was applied to minimize the variance of the lifetime model parameters. They argued the D-optimal design gives the best step stress loading scheme. Moreover, Hu et al. investigated the optimization plans for the step stress levels in SSADT with a Wiener degradation model, and argued that an optimal strategy for SSADT test can only be accomplished with no more than two step stress levels [26].

Recently, there has been a couple of cases of using SSADT to estimate the lifetimes of LED products. Huang et al. built up a SSADT model by using a stochastic Wiener process to analyze the luminous flux data of pc-white LEDs collected from a k-step stress accelerated degradation test [27]. With this model, a 2-step stress accelerated degradation test is proved to give similar lifetime estimation of the pc-white LEDs when compared to CSADT, but halve the sample size. Cai et al. developed a three-step stress accelerated test on the light source of a LED lamp. The test data were further used together with a fault tree and Monte Carlo algorithm to predict the degradation of luminous flux of the LED lamp [28]. 

Although the above-mentioned SSADT models gained a successful use in literatures, it is noted that all of them are empirical models whose parameters were obtained by the curve fitting from experimental results. The physical failure mechanisms that occurred during the whole process of SSADT are unfortunately not fully investigated except for a rough assumption of no additional failure modes. Under such circumstances, the development of an optimum step stress strategy is always cumbersome and time-consuming as a huge amounts of experimental works need to be done. To enhance our understanding the failures existing in the SSADT for pc-white LEDs, a comprehensive study on the failure analysis during the ageing process of SSADT for CSP LEDs, one of the most promising white LED light sources with benefits of small size, high power, and high color quality, is carried on in this paper. The remainder of this paper is organized as follows: Section 2 introduces the test samples used in this study and some conducted experiments. Section 3 discusses the measurement results from experiments. Finally, the concluding remarks are presented in Section 4.

## 2. Test Samples and Experiments

### 2.1. Test Sample Preparation

**Figure 1:**
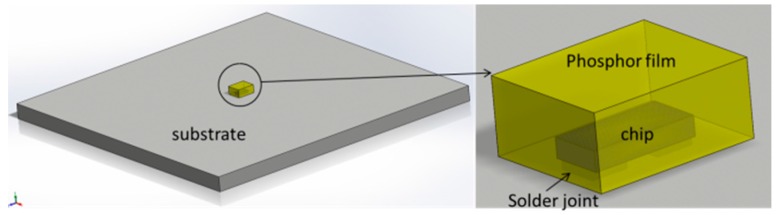
The structure of white chip scale package LEDs (LED CSP).

Nowadays, wafer level chip scale packaging (WLCSP) technology has attracted a lot of attention in manufacturing small-size, low assembly dependent, high color rendering, high thermal and electrical conductivities, and high reliable pc-white LEDs, which are called chip scale package LEDs (CSP LEDs) [29,30]. The CSP LEDs produced by the flip chip process were adopted in this study. As illustrated in Figure 1, this kind of LED has a simple structure that only consists of a phosphor layer, blue LED chip, and solders joints attached to substrate, resulting in relatively simple failure modes during the SSADT process as well. 

**Figure 2:**
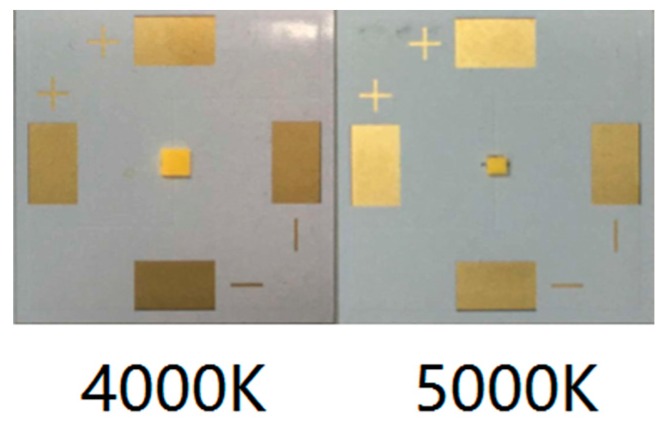
Illustration of the test samples.

**Figure 3:**
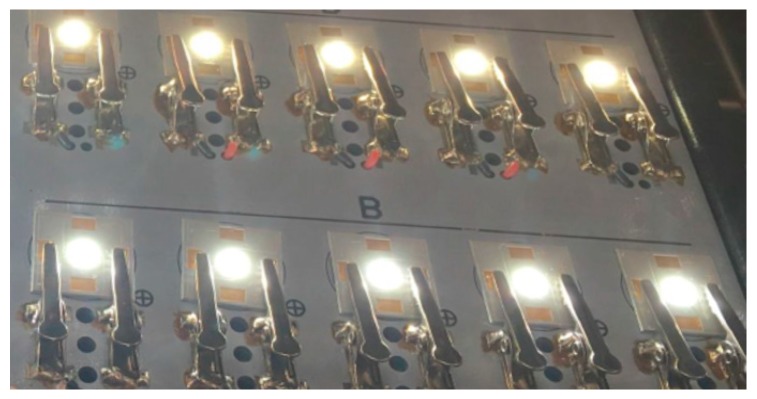
The CSP LED samples under 120 mA during the step-stress accelerated degradation test (SSADT) process. (Top: 4000 °K samples; Bottom: 5000 °K samples).

In this paper, two types of CSP LED samples with different powers were prepared in house with correlated color temperatures (CCTs) of 4000 °K and 5000 °K, respectively, as shown in Figure 2. Information of the prepared samples is given in Table 1, including the phosphor weight ratio, phosphor composition, and sample dimension. Among the phosphor compositions, YAG 04, GM537H, and RH650D are the commercial yellow, green, and red phosphors, respectively. The LED chips were bonded on an aluminum substrate using a eutectic bonding process with the SAC 305 solder paste. Depending on the applied LED chips, the rated currents of driving the CSP LED samples with CCTs of 4000 °K and 5000 °K are 350 mA and 120 mA, respectively. Then, the 4000 °K and 5000 °K samples were further mounted on an aluminum core board by customized aluminum clamps for SSADT, as shown in Figure 3, where the samples were tested under 120 mA during the SSADT process.

### 2.2. Junction Temperature Measurements

The junction temperature of the LED CSP test sample was measured by a linear relationship between junction temperature and voltage in semiconductor packages [31]. The temperature-voltage curves of the test samples in this study were measured by a LEETs LEDT-300B test equipment where its main technical characteristics are listed in Table 2. The temperature-voltage coefficients, also called *K* factors, were then extracted from these temperature-voltage curves. Once the *K* coefficient is obtained, the junction temperature of a CSP LED sample can be calculated based on its driving voltage by Equation (1)

.
(1) $$T_{j}=T_{a}+(V_{j}-V_{a})/K$$

where *T*_a_ is the environmental temperature and *V*_a_ is the voltage of the test sample under a very small driving current, such as 1 mA; *T*_i_ is the junction temperature, and *V*_a_ is the voltage of the test sample under the given driving current. The junction temperature can be further used to estimate the junction-to-air thermal resistance of the test sample by Equation (2).

(2) $$R_{th}=\frac{T_{j}-T_{p}}{P_{th}}=\frac{T_{j}-T_{p}}{P_{tot}-P_{opt}}$$

where *R*_th_ is the junction-to-board thermal resistance, *T*_p_ is the board temperature, *P*_th_ is the sample’s thermal power, calculated by subtracting the optical power from the total electrical power.

### 2.3. Step Stress Accelerated Degradation Test

**Figure 4:**
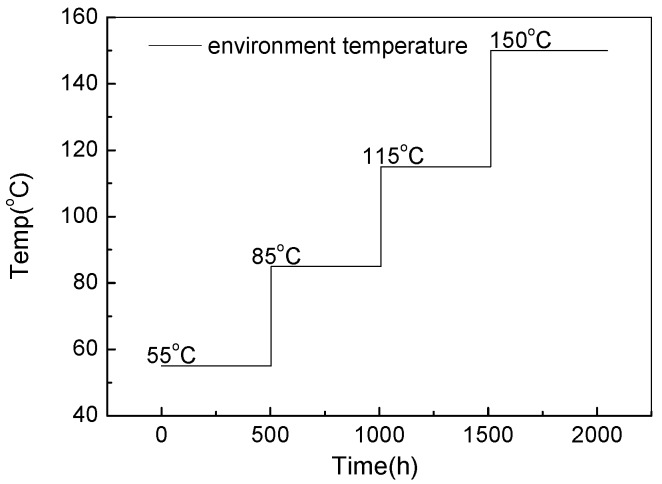
Illustration of the 4-step stress accelerated degradation test.

Determination of the stress levels during the SSADT process obeys the following principles. Firstly, no additional failure modes should be introduced during the whole test process. Secondly, the maximum environmental temperature should be high enough to reduce the test period. Thirdly, the testing time at each stress level should be long enough with a great exposure to the potential failure risk. When considering all of the above-mentioned issues, the environmental temperature was set from 55 °C to 150 °C, with an increment of 30 °C in every 504 h, as illustrated in Figure 4, in SSADT. During the test process, the LED CSP test samples were divided into two groups each of which contains 5 samples, under driving currents of 350 mA and 120 mA, respectively. Note that, the 5000 °K sample under a driving current of 350 mA is in about three times overdrive since its rated current is only 120 mA. Once in a while, the test was paused to measure the photometric and chromatic properties, such as spectral power distributions (SPD), luminous flux, color coordinates, and CCT of the test samples using a 0.5 m integrating sphere at room temperature. In total, there are 10 times measurements, each of which the stoppage time is less than 8 h.

## 3. Results and Discussion

### 3.1. Thermal Analysis

**Figure 5:**
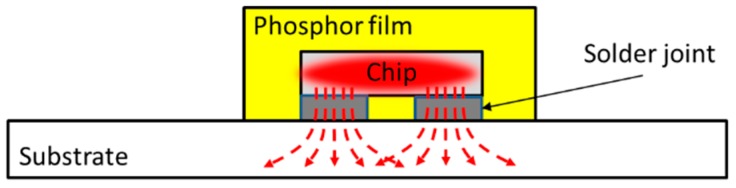
Illustration of the heat transfer path in a CSP LED.

As shown in Figure 5, most of the heat generated by the chip is dissipated through the LED body, the dissipation efficiency relies on thermal properties of all composing materials and interface along the heat transfer path. During the SSADT process, degradation of the thermal properties of the test sample usually appears concurrently with degradation of the photometric and chromatic properties of the CSP LED samples. Early tests on LEDs under 85 °C/85RH conditions showed that the LED junction temperature increases continuously along with the progress of the test due to a moisture induced delamination between the chip and lead frame [32]. A similar junction temperature rising phenomenon is also observed in our experiments. 

**Figure 6:**
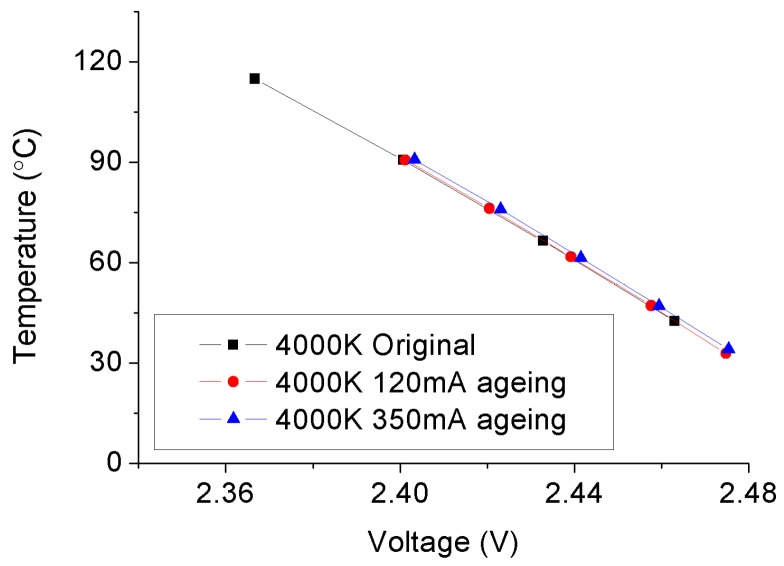
Temperature-voltage curves of the 4000 °K samples before and after SSADTs with 2046 h.

**Figure 7:**
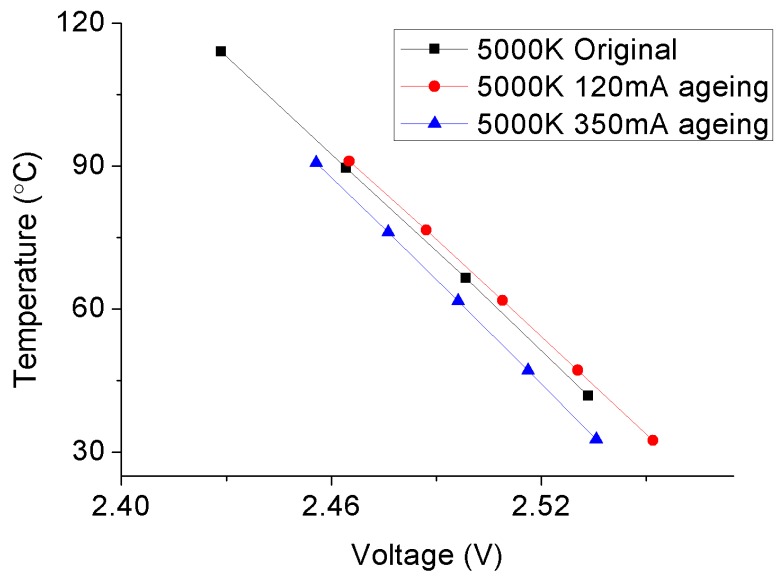
Temperature-voltage curves of the 5000 °K samples before and after SSADT with 2046 h.

The temperature-voltage curves of typical 4000 °K and 5000 °K samples before and after SSADTs under 120 mA and 350 mA are plotted in Figure 6 and Figure 7 for comparison. These curves show the linear relationships between the junction temperature and voltage driving under 1 mA of the test samples. 

As shown in Figure 6, the temperature-voltage curves of the 4000 °K sample before and after SSADT remain the same under a driving current of 120 mA. Even driven by a current of 350 mA, the temperature-voltage curve only shifts slightly to the right when compared to that measured before SSADT. As a result, it can be argued that it will not introduce extra failures by elevating the driving current from 120 mA to 350 mA.

**Figure 8:**
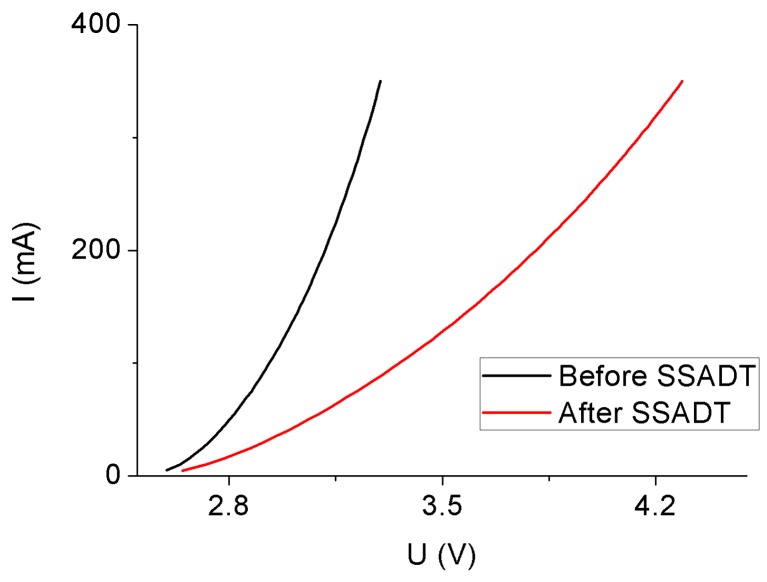
Current-voltage curves of the 5000 °K samples before and after SSADT.

For the 5000 °K sample, a significant driving current effect is exhibited on the temperature-voltage curves, as shown in Figure 7. Similar to the 4000 °K sample, the temperature-voltage curve is slightly shifted to the right under its rated current which is 120 mA. However, by increasing the driving current to 350 mA, an apparently opposite shift is found in between the temperature-voltage curves before and after SSADT. This might be because that, under a long-term over-driving condition, the active layer of the LED chip is partially damaged to reduce both the optical and heat output of the 5000 °K sample. Figure 8 plots the I-V curves of the 5000 °K sample under a driving current of 350 mA before and after SSADT. It can be seen that after SSADT, the 5000 °K sample exhibited a “flat” I-V curve which can be regarded by connecting a series resistance induced from the damage in the active layer [33]. 

Based on the temperature-voltage curves plotted in Figure 6 and Figure 7, the junction temperatures of test samples before and after SSADT were calculated by Equation (1). The calculation process is divided into three steps: Firstly, a driving current (i.e., 350 mA for the 5000 °K samples and 120 mA for the 4000 °K samples) was applied on the test sample for 30 minutes that is long enough to reach a steady-state temperature distribution over that sample; then, the driving current was turned off, and immediately a 1 mA current was applied instead; lastly, the voltage of the sample was measured to calculate the junction temperature by using Equation (1), in which the K coefficient was extracted from the plots shown in Figure 6 and Figure 7. Subsequently, the junction-to-board thermal resistances were further calculated by Equation (2), and enumerated in Table 3 with the calculated junction temperatures and other parameters used in Equation (2). According to Table 3, the junction temperature of the 4000 °K sample continuously increases with the rise of the driving current. This implies that the driving current acts the same with the temperature stress on the degradation of the 4000 °K samples. However, as to the 5000 °K sample, its junction temperature increases under the driving current of 120 mA, but drop obviously when the driving current is lifted up to 350 mA. Such a reduction is because of the additional failures on the 5000 °K sample that was caused by the over-driving current. In addition, a relatively higher increase rate on board temperatures of both 4000 °K and 5000 °K samples (except for those driven under 350 mA) was observed as compared to the junction temperatures. This makes the junction-to-board thermal resistances of these samples show a continuous decrease during SSADT. As to the 5000 °K samples after SSADT driven under 350 mA, the junction-to-board thermal resistances were dramatically reduced to about 1/3 of its original value, proving that the LED chip might be seriously damaged by the over-driving current.

### 3.2. Spectral Power Distribution Attenuation

**Figure 9:**
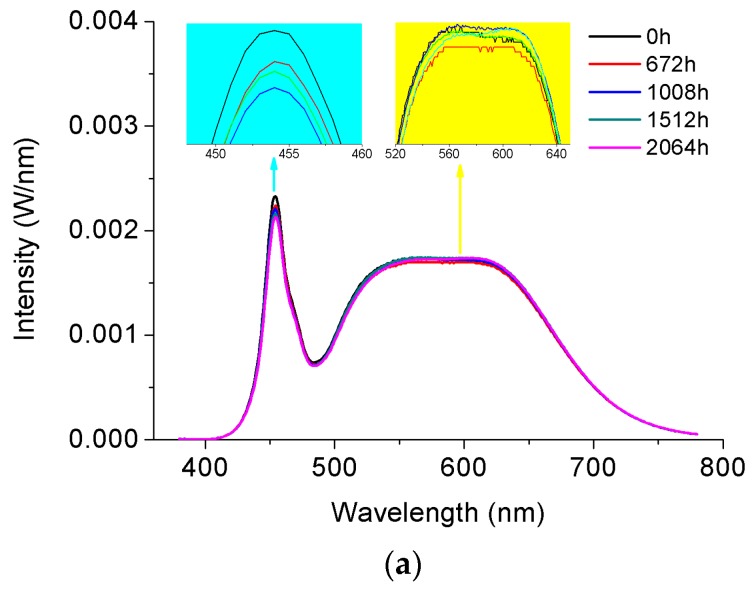

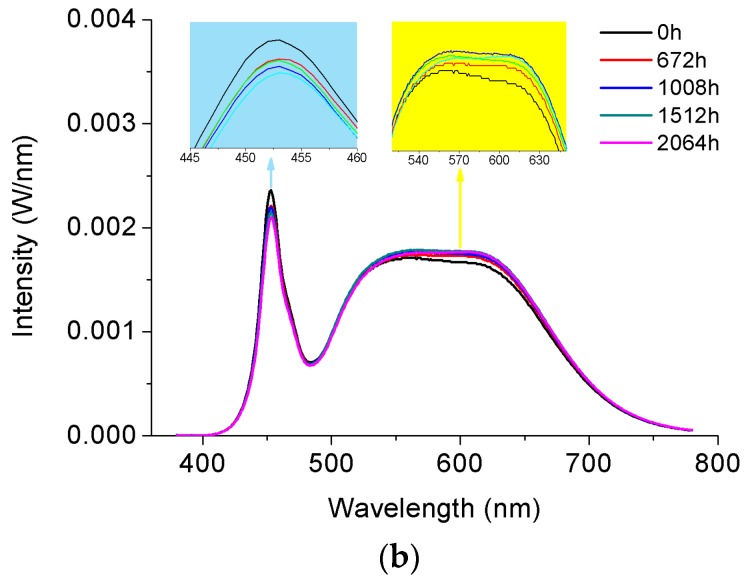
Attenuation of spectral power distributions of the 4000 °K samples during SSADT. (**a**) Driving by a 120 mA current; (**b**) Driving by a 350 mA current.

**Figure 10:**
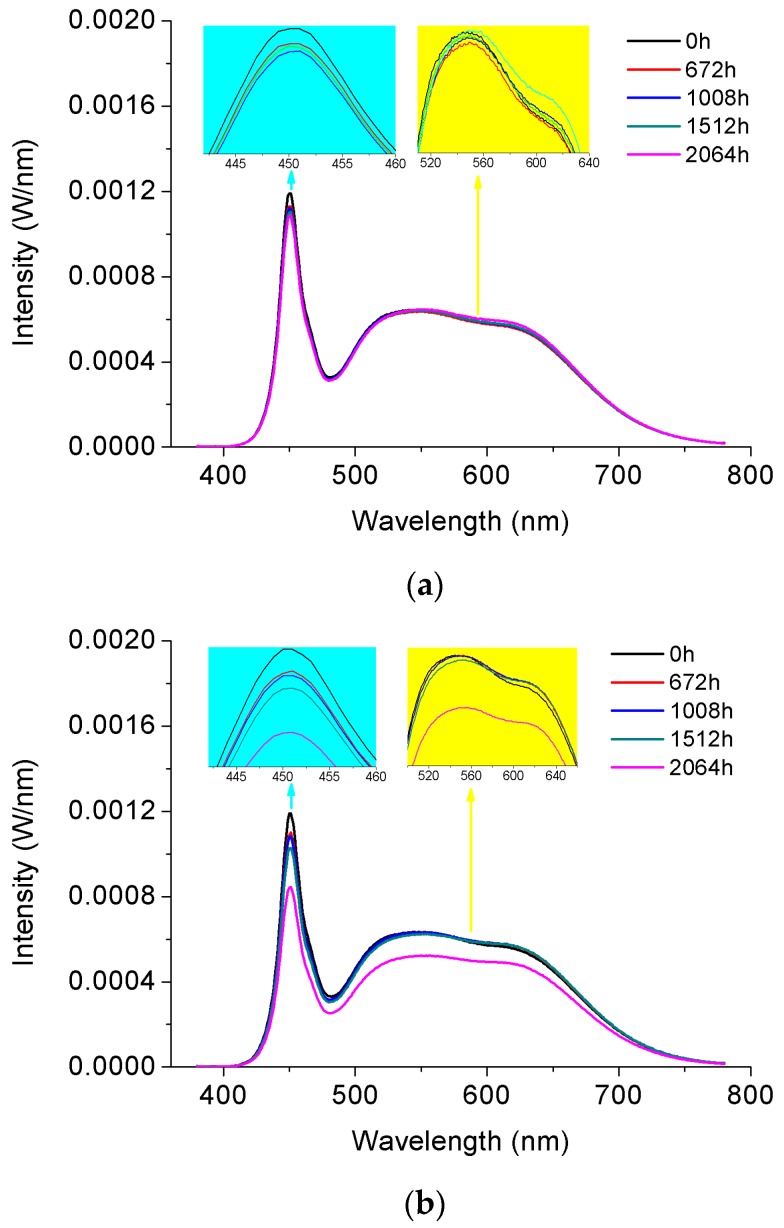
Attenuation of spectrum power distributions of the 5000 °K samples during SSADT. (**a**) Driving by a 120 mA current; (**b**) Driving by a 350 mA current.

Figure 9 and Figure 10 reveal the attenuation of SPDs of the test samples by plotting a number of SPDs of randomly selected 4000 °K and 5000 °K samples measured in about every 504 h of SSADT under the driving currents of 120 mA and 350 mA, respectively. As shown in Figure 9a, with the increase of the ageing time, the blue peak of the SPD is slightly weakened, whereas the phosphor converted peak shows a fluctuating downward trend. This implies that degradation of the LED chip and phosphor is evolved with different failure mechanisms. During SSADT, the LED chip is continuously degraded, corresponding to the rise of junction temperature, as shown in Table 3. However, the degradation of phosphor is not explicit due to the mutual synergistic effect of different thermal quenching failure mechanisms of its material constitutions [34].

The time-varying SPDs of the 4000 °K sample during SSADT under a driving current of 350 mA are plotted in Figure 9b. It can be seen that the SPDs shown in Figure 9b follow the same but more exaggerated attenuation trend as compared to those shown in Figure 9a. This agrees with the observations from the temperature-voltage curves shown in Figure 6 that no additional failure is occurred by increasing the driving current from 120 mA to 350 mA.

The SPD attenuation is not altered for the 5000 °K sample under 120 mA during SSADT, as shown in Figure 10a, and under 350 mA during the first 1500 h of SSADT, as shown in Figure 10b. However, a dramatic fall is observed all over the SPD of the 5000 °K sample after 1500 h ageing under 350 mA. This supports the argument that the LED chip has been seriously damaged by such a high driving current after a long term of SSADT, as discussed in the aforementioned subsection.

### 3.3. Lumen Maintenance Depreciation

**Figure 11:**
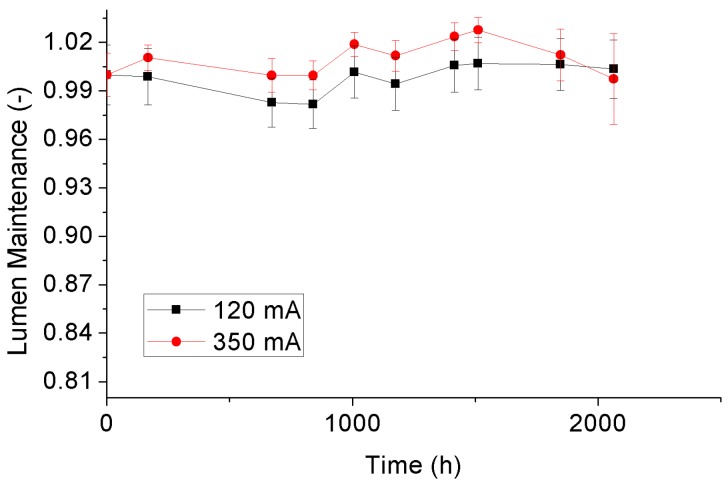
Lumen maintenance depreciation of the 4000 °K samples.

**Figure 12:**
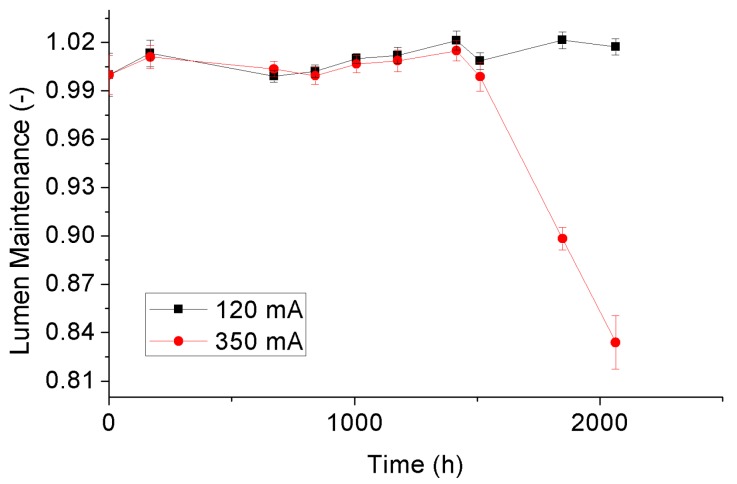
Lumen maintenance depreciation of the 5000 °K samples.

Luminous fluxes of the test samples at a certain ageing time can be calculated based on their SPDs at that time. Then, by normalizing these luminous fluxes with the average of the initial values at 0 h, the lumen maintenances can be further determined. Figure 11 and Figure 12 display the variance trends of the averaged lumen maintenances with statistical error bars of the 4000 °K and 5000 °K samples, respectively. It can be seen that lumen maintenances of the 4000 °K and 5000 °K tests samples under a driving current of 120 mA are not presented downward trends during the whole SSADT process, indicating that they are still in a seasoning state. Driving by a current of 350 mA, the 4000 °K and 5000 °K tests samples show a same trend on lumen maintenances in the first 1500 h. After that, both test sample behave degradation under the dual effect of high temperature and high current. When compared to the 4000 °K samples, the 5000 °K samples, of which the rated current is far less than 350 mA, expressed a much rapid lumen maintenance depreciation that is caused by LED chip damage. This implies the application of a high driving current is helpful to accelerate the lumen maintenance depreciation of the test samples. However, an extreme over-drive current should be avoided since it increases the risk of chip damage to a great extent.

### 3.4. Color Shift

**Figure 13:**
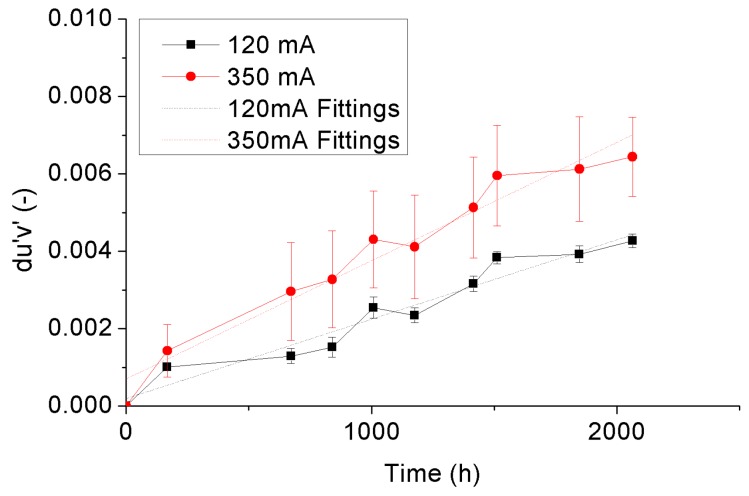
Color shift of the 4000 °K samples.

**Figure 14:**
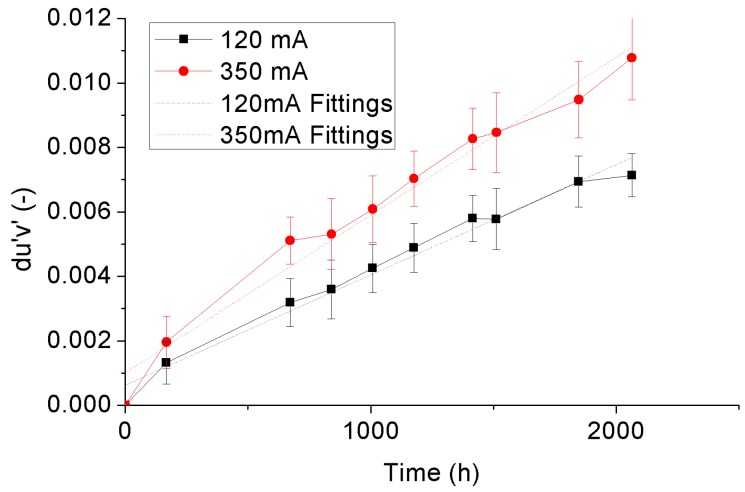
Color shift of the 5000 °K samples.

Color shift is a LED reliability characteristic related to the deviation of color coordinates from the initial values, and therefore can be calculated by Equation (3).

(3) $$du^{\prime }v^{\prime }=\sqrt{{\left(u^{\prime }-{u^{\prime }}_{0}\right)}^{2}+{\left(v^{\prime }-{v^{\prime }}_{0}\right)}^{2}}$$

in which *u*’ and *v*’ are color coordinates in CIE 1976 color space, and *u*_0_’ and *v*_0_’ are the initial values of the color coordinates [35,36,37,38,39]. Figure 13 and Figure 14 display the variance trends of the averaged *du*’*v*’ with statistical error bars of the 4000 °K and 5000 °K samples, respectively. An overall view from these two figures is that the *du*’*v*’ of both 4000 °K and 5000 °K samples show linear growing trends with the increase of ageing time, in which the growth rate is highly dependent on the driving current. Equation (4) is applied to describe the growing curve of *du*’*v*’ of the test samples, and the corresponding fitting curves to the 4000 °K and 5000 °K samples are also plotted with the dotted lines in Figure 13 and Figure 14, respectively.

(4) $$du^{\prime }v^{\prime }=C_{1}\times t+C_{2}$$

in which *C*_1_ is the growth rate, *C*_2_ is the intercept, and *t* is the ageing time.

Table 4 enumerates the fitting coefficients in Equation (3) of the 4000 °K and 5000 °K samples under driving currents of 120 mA and 350 mA. It can be seen that the growth rates of the 4000 °K and 5000 °K samples aged under each own rated current are similar. The growth rate will move synchronously with the adjustment of the driving current. For quantitatively qualifying the current effect on color shift of the test samples, the acceleration factors in between both samples were calculated based on the growth rates of them by Equation (5), and shown in Table 4 as well.

(5) $$AF_{du^{\prime }v^{\prime }}=C_{1,350\mathrm{mA}}/C_{1,120\mathrm{mA}}$$

in which *AF_du’v’_* is the acceleration factor of the growth rate of color shift, *C*_1,350 mA_ and *C*_1,120 mA_ are the growth rates of the test samples aged under currents of 350 mA and 120 mA, respectively.

**Figure 15:**
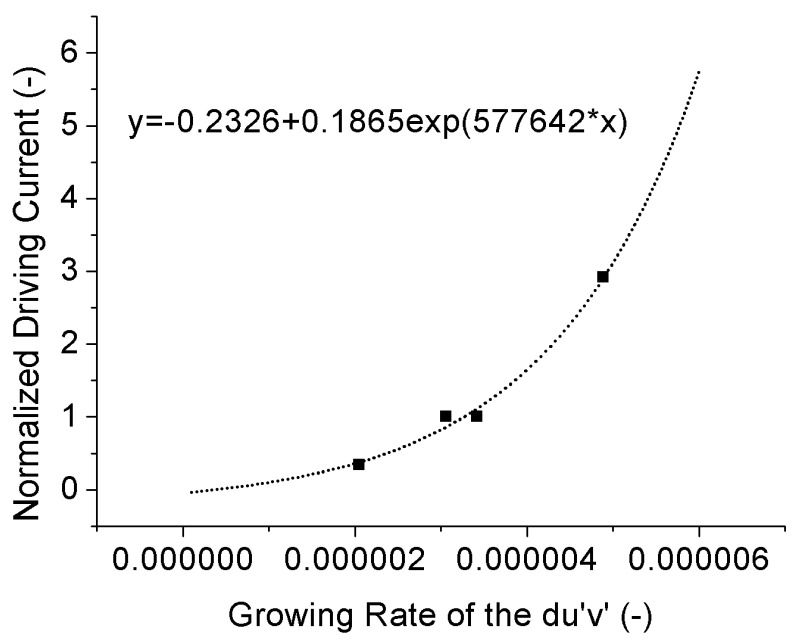
Current effect on the growth rate of the *du’v’*.

As seen in Figure 13, Figure 14 and Table 4, by increasing the driving current from 120 mA to 350 mA, the growth rates of *du*’*v*’ of both 4000 °K and 5000 °K samples are increased with similar acceleration factors which are independent on the rated currents of the test samples. Therefore, it allows us to normalize the driving currents of both 4000 °K and 5000 °K samples by their rated currents to give an overall view of the driving current effect on *du*’*v*’ of the test samples, as shown in Figure 15. This driving current effect is possible to be fitted by a three-parameter exponential curve in which the offset, pre-factor and power factor are −0.2326, 0.1865 and 577,642, respectively, as shown in Figure 15 as well.

### 3.5. CCT Depreciation

**Figure 16:**
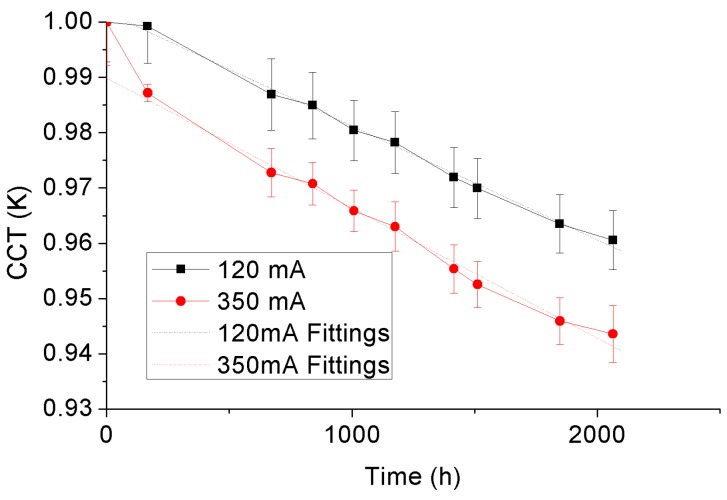
CCT depreciation of the 4000 °K samples.

**Figure 17:**
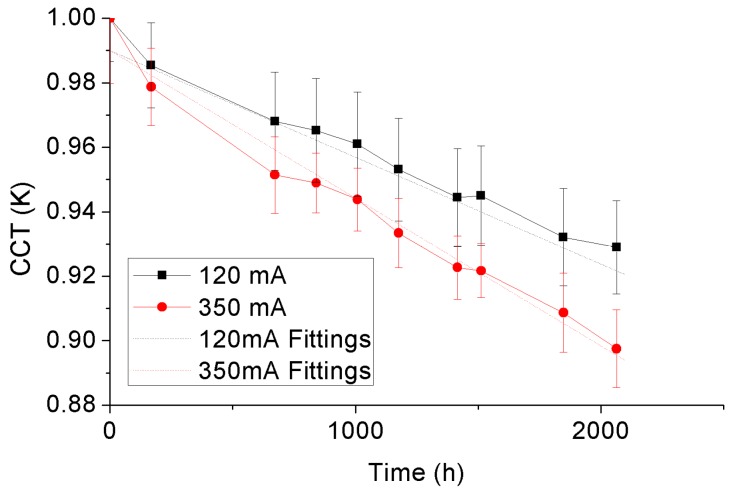
CCT depreciation of the 5000 °K samples.

The correlated color temperature (CCT) represents the corresponding blackbody locus temperature of a pair of color coordinates along the iso-temperature line. Therefore, during SSADT, the CCT will show a time-varying variance along with the change of color coordinates. Figure 16 and display the CCTs of the 4000 °K and 5000 °K samples with statistical error bars during SSADT. For the purpose of comparison, CCTs of each of the test samples are normalized by its initial values. According to Figure 16 and Figure 17, the CCTs of both 4000 °K and 5000 °K samples show linear degradation trends with the ageing time, opposite to the varying trends of *du*’*v*’. This linear degradation trend of CCTs was also found in other scenarios, for instance, from high power LED lamps under an 85 °C/85% relative humidity test [40]. To describe this linear degradation trend, Equation (6) is applied in our study, and the acceleration factor of the degradation rates extracted from two different driving currents is calculated by Equation (7).

(6) $$CCT=D_{1}\times t+D_{2}$$

(7) $$AF_{CCT}=D_{1350\mathrm{mA}}/D_{1120\;\mathrm{mA}}$$

in which *D*_1350 mA_ is the degradation rate, *D*_2120 mA_ is the intercept, *t* is the ageing time, *AF_CCT_* is the acceleration factor, *D*_1350 mA_, and *D*_1120 mA_ are the degradation rates of the test samples aged under currents of 350 mA and 120 mA, respectively.

Table 5 enumerates the fitting parameters in Equation (6) of the 4000 °K and 5000 °K samples under driving currents of 120 mA and 350 mA, and the resulting acceleration factors of the degradation rates. From Table 5, it can be seen that the driving current also plays an important role on the degradation of CCTs for both test samples. Such an effect is found relatively more significant on the 5000 °K samples.

## 4. Conclusions

In this paper, the reliability issues, including thermal, photometric, and colorimetric properties, of LED chip scale packages during SSADT with driving currents of 120 mA and 350 mA were thoroughly investigated. The ageing process of SSADT is composed by four temperature stages from 55 °C to 150 °C, each step lasts 504 h. Two different types of LED chip scale packages were tested. The rate current is 350 mA for the 4000 °K samples, and 120 mA for the 5000 °K samples.

The thermal properties of the test samples were investigated via the comparison of junction temperatures and the temperature-voltage curves before and after SSADT. For a sample driving by a current below or equaling to the rated current, its junction temperature after SSADT was increased to a certain extent depending on the driving current. However, for the 5000 °K sample overdriven by a current of 350 mA, the junction temperature after SSADT was reduced, owing to a left shift of the temperature-voltage curve. This might be caused by partial damage in the active layer of the LED chip induced by a synergistic effect of the high temperature and overdrive current. The supporting evidence was obtained from two aspects: firstly, an obvious series resistance existed in the I-V curve of the 5000 °K sample after SSADT; secondly, a dramatic SPD attenuation of the 5000 °K sample was observed in the end of SSADT. 

Finally, the time-dependent variances of lumen maintenance, color shift, and CCT of the test samples during SSADT, and the driving current effect on these results were studied in detail. The results suggest that failures, such as color shift and CCT depreciation of the LED chip scale packages, can be effectively aroused by SSADT. The varying rates of these two failures are further proved to have a positive relationship with the driving current.

At last, it is worth mentioning that similar time-varying trends of the photometric and colorimetric parameters observed in this study would be likely to occur in all types of phosphor converted LEDs, since the failure mechanisms among them are not significantly different. More case studies on other type of phosphor converted LEDs will be investigated in future studies to explore the applicability and usability of SSADT in fast evaluation of the reliability of LEDs.

## Figures and Tables

**Table 1:** Information of the CSP LED samples.

Target CCTs	Phosphor Weight Ratio %	Phosphor Composition (YAG04: GM537H5: RH650D)	Sample Dimension (L × W × H)
4000 °K	18.5	3.2:12.6:4.8	0.97 mm × 0.97 mm × 0.15 mm
5000 °K	18.5	0:3.38:1	0.78 mm × 0.38 mm × 0.14 mm

**Table 2:** Technical characteristics of the LEETS LEDT-300B test equipment.

Item	Description
Manufacturer	LEETs Lighting Shanghai
Rated voltage	220 V 50/60 Hz
Operational current	<0.5 A
Junction temperature measurement current	1 mA/5 mA ± 0.2%
Temperature tolerance of K coefficient	±0.5 °C
Sampling pulse width	5 ms
Junction temperature measurement error	±1 °C

**Table 3:** Thermal properties of test samples before and after SSADT.

Driving Conditions	4000 °K Sample				5000 °K Sample			
	*T*_j_ (°C)	*T*_p_ (°C)	*P*_th_ (W)	*R*_th_ (°C/W)	*T*_j_ (°C)	*T*_p_ (°C)	*P*_th_ (W)	*R*_th_ (°C/W)
Original	75.52	48.08	0.710	38.63	54.01	34.37	0.219	89.84
After test under 120 mA	84.38	58.59	0.744	34.65	59.78	44.66	0.212	71.39
After test under 350 mA	89.41	69.01	0.745	27.37	51.27	41.99	0.297	31.24

**Table 4:** Parameters of the linear color shift model.

Driving Conditions	Parameters	4000 °K Sample	5000 °K Sample
Driving by 120 mA	*C*_1,120 mA_	2.05 × 10^−6^	3.42 × 10^−6^
	*C*_2,120 mA_	1.94 × 10^−4^	6.28 × 10^−4^
Driving by 350 mA	*C*_1,350 mA_	3.06 × 10^−6^	4.89 × 10^−6^
	*C*_2,350 mA_	7.03 × 10^−4^	1.02 × 10^−3^
*AF_du’v’_*		1.49	1.43

**Table 5:** Parameters of the linear CCT model.

Driving Conditions	Parameters	4000 °K Sample	5000 °K Sample
Driving by 120 mA	*D*_1120 mA_	−2.05 × 10^−5^	−3.05 × 10^−5^
	*D*_2120 mA_	1.002	0.990
Driving by 350 mA	*D*_1350 mA_	−2.35 × 10^−5^	−4.14 × 10^−5^
	*D*_2350 mA_	0.990	0.983
*AF_CCT_*		1.15	1.36

## Abbreviations

CCT	Correlated Color Temperature
CSP	Chip Scale Package
CSADT	Constant-Stress Accelerated Degradation Test
LED	Light-emitting Diode
SPD	Spectral Power Distribution
SSADT	Step-Stress Accelerated Degradation Test
WLCSP	Wafer Level Chip Scale Packaging
